# c-Met is expressed by highly autoreactive encephalitogenic CD8+ cells

**DOI:** 10.1186/s12974-019-1676-0

**Published:** 2020-02-19

**Authors:** Mahdia Benkhoucha, Isis Senoner, Patrice H. Lalive

**Affiliations:** 1 Department of Pathology and Immunology, Faculty of Medicine, University of Geneva, Geneva, Switzerland; 2 Department of Neurosciences, Division of Neurology, University Hospital of Geneva, Geneva, Switzerland

**Keywords:** HGF, c-Met, CD8^+^ T cell, Neuroinflammation, EAE, MS

## Abstract

**Background:**

CD8^+^ T lymphocytes are critical mediators of neuroinflammatory diseases. Understanding the mechanisms that govern the function of this T cell population is crucial to better understanding central nervous system autoimmune disease pathology. We recently identified a novel population of highly cytotoxic c-Met-expressing CD8^+^ T lymphocytes and found that hepatocyte growth factor (HGF) limits effective murine cytotoxic T cell responses in cancer models. Here, we examined the role of c-Met-expressing CD8^+^ T cells by using a MOG_35–55_ T cell-mediated EAE model.

**Methods:**

Mice were subcutaneously immunized with myelin oligodendrocyte glycoprotein peptide (MOG)_35–55_ in complete Freund’s adjuvant (CFA). Peripheral and CNS inflammation was evaluated at peak disease and chronic phase, and c-Met expression by CD8 was evaluated by flow cytometry and immunofluorescence. Molecular, cellular, and killing function analysis were performed by real-time PCR, ELISA, flow cytometry, and killing assay.

**Results:**

In the present study, we observed that a fraction of murine effector CD8^+^ T cells expressed c-Met receptor (c-Met^+^CD8^+^) in an experimental autoimmune encephalitis (EAE) model. Phenotypic and functional analysis of c-Met^+^CD8^+^ T cells revealed that they recognize the encephalitogenic epitope myelin oligodendrocyte glycoprotein_37–50_. We demonstrated that this T cell population produces higher levels of interferon-γ and granzyme B *ex vivo* and that HGF directly restrains the cytolytic function of c-Met^+^CD8^+^ T cells in cell-mediated cytotoxicity reactions

**Conclusions:**

Altogether, our findings suggest that the HGF/c-Met pathway could be exploited to modulate CD8^+^ T cell-mediated neuroinflammation.

## Introduction

The prevailing opinion is that multiple sclerosis (MS) is a CD4 T cell-mediated disease. However, many indicators imply a role for CD8^+^ T cells in MS pathophysiology, including their presence in a greater number than CD4^+^ T cells in MS-related brain lesions and a demonstration of direct axonal toxicity [1–6]. The exact role of CD8^+^ T lymphocytes in autoimmune central nervous system (CNS) inflammation remains controversial, and some evidence supports both pathogenic and protective roles for these cells in MS and in experimental autoimmune encephalomyelitis (EAE). In favor of the pathogenic contribution of CD8^+^ T lymphocytes, oligoclonally expanded CD8^+^ T cells have been detected in demyelinated MS tissue [4], suggesting CNS antigen (Ag) reactivity and CD8^+^ T cells dependent lytic functions. CD8^+^ T cells also have been found in close association with demyelinated axons in MS brain tissue [7] and interact with neural cells [8]. Other groups have developed various models of CD8^+^ T lymphocyte-dependent EAE-like disease [9–13]. These models include pathogenesis induction by transfer of myelin basic protein_79–87_ or myelin oligodendrocyte glycoprotein (MOG)_35–55_-specific CD8^+^ T lymphocytes in C3H and in C57BL/6 or RAG1/animals, through the use of MHC tetramer technology. The characterization of MOG_35–55_-specific CD8^+^ T cells throughout EAE progression showed that these cells are generated in MOG_35–55_-induced EAE mice in vivo and that MOG_37–50_ retains the full ability to stimulate MOG-specific CD8^+^ T cells in vitro [14].

Several reports describe a role for encephalitogenic CD8^+^ T cells in EAE. A protocol using extracellular vesicle fibrinogen administered to mice with EAE at peak clinical disease resulted in a unique spontaneous phenotype of relapsing−remitting disease, a clinical feature not generally observed in this MOG_35–55_ EAE mouse model [15]. In addition, CD8^+^ T cells can mediate demyelination and oligodendrocyte toxicity through secretion of toxic factors, such as lymphotoxin [16, 17]. Human and mouse CD8^+^ T lymphocytes detected in the inflamed CNS compartment are predominantly CD62L^-^ CCR7^-^ effector memory lymphocytes. Compared to non-effector memory cells, these cells have a greater propensity to migrate across the human blood–brain barrier. In EAE, CD8^+^ T lymphocytes infiltrating the CNS display more aggressive functions (granzyme B^+^ interferon [IFN]-γ^hi^) than do CD4^+^ T lymphocytes [18].

Conversely, other studies have suggested a protective role for CD8^+^ T cells in both EAE and MS [19, 20]. CD8^+^ T lymphocytes can modulate the phenotype of CD4^+^ T lymphocytes in the periphery of EAE mice by increasing Th2 cells [21]. In a myelin basic protein-induced EAE model, CD8^+^ T lymphocytes downregulated pathogenic myelin basic protein-reactive CD4^+^ T lymphocyte clones [22]. In addition, the transfer of MOG_35–55_-specific CD8^+^ T lymphocytes in mice suppresses the induction of EAE and inhibits ongoing EAE by a cytotoxic/suppressor mechanism [23, 24]. Therefore, the precise contribution of CD8^+^ T lymphocytes to the pathology of EAE and MS remains unclear.

The hepatocyte growth factor (HGF)/c-Met axis modulates several inflammatory-mediated diseases by acting on a wide variety of cells [25], including in EAE [26, 27]. Recent reports have stressed the multiple anti-inflammatory effects of HGF, including a Th2/Th3 bystander deviation with increased transforming growth factor (TGF)-β1 and interleukin (IL)-10 [28, 29] and inhibition of Ag-presenting cell (APC) function [26, 28, 30, 31]. HGF may also contribute to the expansion of myeloid-derived suppressor cells, which are potent T cell suppressors [32]. All of these effects could contribute to the protective action of HGF in EAE and/or MS either individually or collectively.

We recently found that a fraction of murine cytotoxic lymphocytes (CTLs) expresses c-Met (c-Met^+^ CTLs). Functional and phenotypic analysis of this newly identified CTL population showed that c-Met^+^ CTLs display augmented cytolytic activities compared to their c-Met^−^ CTL counterparts. Using cell-mediated cytotoxicity reactions in vitro and in vivo, we demonstrated also that the c-Met receptor ligand HGF can directly negatively regulate c-Met^+^ CTL cytolytic function in a cancer model [33].

In this study, we examined the role of c-Met-expressing CD8^+^ T cells by using a MOG_35–55_ T cell-mediated EAE model. Our aim was to complement previous studies of the function of the HGF/c-Met pathway in “conventional” CD4^+^ T cell-mediated demyelination [26, 34]. We found that a subpopulation of effector CD8^+^ T cells expresses c-Met in EAE. In addition, our results offer a phenotypic and functional characterization of this population ex vivo and in vitro. We also examined the capacity of the c-Met ligand HGF to modulate c-Met-expressing CD8^+^ T cell function. Our findings suggest that c-Met expression by CD8^+^ T cells confers specific pro-inflammatory properties in MOG_35–55_-induced EAE.

## Materials and methods

### Mice

C57BL/6 J (H-2^b^) female mice were purchased from Charles River (France). Animals were housed in a specific pathogen-free barrier facility at the Medical Center of Geneva, Faculty of Medicine (Geneva, Switzerland). All experimental protocols and procedures were reviewed and approved by the Institutional Animal Care and Use Committee of the Geneva University School of Medicine. Animal care and experimental procedures were carried out in accordance with the guidelines of the Institutional Animal Care and Use Committee of the Geneva University School of Medicine.

### Induction and assessment of EAE

Ten mice were immunized subcutaneously in the flanks with 200 μg MOG_35–55_ peptide (MEVGWYRSPFSRVVHLYRNGK; Anawa Trading) in complete CFA (DIFCO Laboratories), and 300 ng pertussis toxin (Sigma-Aldrich) in phosphate-buffered saline (PBS) was administered intravenously on days 0 and 2. Individual animals were observed daily, and clinical scores were assessed with a 0–5 point scoring system as follows: 0 = no clinical disease, 1 = loss of tail tone only, 2 = mild monoparesis or paraparesis, 3 = severe paraparesis, 4 = paraplegia and/or quadraparesis, and 5 = moribund or death. Mice exceeding 2 days with a score of 3.5 were euthanized immediately. All analyses were performed on mice at peak disease reaching an EAE score of 2 and above.

### CNS cell isolation

CNS mononuclear cells were isolated from EAE mice at different time points (non-immunized mice d0, peak disease d14, and at recovery phase d24) after cardiac perfusion with PBS. Briefly, minced CNS (spinal cord and brain) tissues were digested with collagenase D (2.5 mg/ml; Roche Diagnostics, Indianapolis, IN) at 37 °C for 60 min. Mononuclear cells were isolated by discontinuous Percoll gradient (70/30%) (Sigma-Aldrich) centrifugation. Lymphocytes were collected from the 30:70% interface and washed. Total cell numbers were determined by counting on a hemocytometer, and viability was assessed by trypan blue exclusion. To exclude the possibility of generating artifacts during the isolation process of CNS-infiltrating cells, the percentages rather than the “absolute” cell numbers were displayed to facilitate comparison of results.

### Flow cytometric analysis

Single-cell suspensions from spleens, lymph nodes, and CNS from d0, d14, and d24 were incubated in blocking solution (PBS with 1% fetal calf serum) for 20 min on ice prior to staining to block nonspecific Fc-mediated interactions. They then were stained for 30 min at 4 °C with FITC, PE, PerCP-Cy5, or allophycocyanin fluorochromes conjugated with antibodies (Abs; 1:100) against CD3, CD8, CD45, c-Met, CD44, CD62L, or appropriate fluorochrome-conjugated, isotype-matched irrelevant Abs to establish background fluorescence. For intracellular cytokine and molecular staining of IFNγ, tumor necrosis factor (TNF), granzyme B, Eomes, Runx3, and Ki67 (see Table 1), T cells were stimulated with PMA (50 ng/ml) plus ionomycin (500 ng/ml) in the presence of brefeldin A (10 μg/ml; Sigma-Aldrich) and then fixed and permeabilized using BD Cytofix/Cytoperm Plus Kit (BD Biosciences). Samples were processed on a FACS Gallios flow cytometer (BD Biosciences) and analyzed using FlowJo analysis software (version 10.3). Live, apoptotic, and dead populations were defined on the basis of 7-AAD Viability Staining Solution from eBioscience according to the manufacturer’s instructions. To determine the viability of cells prior to the fixation and permeabilization, we used the LIVE/DEAD™ Fixable Aqua Dead Cell Stain Kit (Thermo Fisher Scientific).

**Table 1:** Antibodies for flow cytometry

Specificity	Clone	Supplier
CD45R	RA3-6B2	eBioscience
CD3	UCHT1	Biolegend
CD8	53-6.7	eBioscience
CD44	IM7	eBioscience
CD62L	MEL-14	eBioscience
c-Met	eBioclone7	eBioscience
IFN-γ	XMG1.2	eBioscience
Granzyme B	NGZB	eBioscience
TNF	MP6-XT22	eBioscience
Eomes	Dan11mag	eBioscience
Runx3	R3-5G4	BD Biosciences
Ki67	SolA15	eBioscience

### Preparation and treatment of splenic and LN CD8^+^ T cells ex vivo

For bulk cultures, single-cell suspensions were prepared from spleens of EAE mice (at peak disease d14). Briefly, and LN were gently homogenized and passed through a 70-μm nylon cell strainer (Falcon, BD Biosciences). Red blood cells were lysed with ACK lysing buffer (BioWhittaker). Mononuclear cell populations were counted, suspended in culture medium at 1 × 10^6^ cells/ml, and then exposed to mrHGF (30 ng/ml) or PBS (vehicle) for the same duration in the presence of adequate peptide (20 μg/ml of MOG_35–55_ or MOG_37–50_ or gp100_25–33_ control peptide); IL-2 (20 pg/ml; PeproTech) was added to cultures for 48 h prior to performing FACS analysis. In some experiments, EAE spleen cells were harvested; stained at 4 °C for 30 min with CD3, CD8, and c-Met; and purified as CD8^+^c-Met^−^ and CD8^+^c-Met^+^ by cell sorting using a FACS Aria SORP II cell sorter (BD Biosciences). Live cells were gated based on forward scatter and side scatter and by DAPI dye exclusion. Sorted ex vivo c-Met^−^ and c-Met^+^CD8^+^ T cell populations showed similar physical properties of size and internal complexity. Sorted CD8^+^ T cells were incubated with irradiated syngeneic splenocytes loaded with 20 μg/ml (MOG_35–55_, MOG_37–50_, or gp100_25–33_ control peptide) for 48 h in the presence of 20 pg/ml of IL-2.

Human gp100 [33] is a fragment of human melanoma antigen gp100. The gp100-specific, H-2Db-restricted, CD8^+^ T cells are capable to recognize B16 melanoma but not normal melanocytes. Since this peptide is commonly used as an immunogen to test CD8^+^ T cell-specific response in several animal models [33], we decided to use this peptide as control.

### Measurement of effector molecules and cytokines

Granzyme B, IFNγ, and TNF levels were detected in culture supernatants from EAE (d14) T cells from the spleen and CNS and tissues using ELISA sets from eBioscience or R&D Systems according to the respective manufacturer’s instructions.

### Ex vivo cytotoxicity assay

The in vitro killing activities of splenic CD8^+^ T cells obtained from MOG_35–55_-induced EAE (d14) stimulated ex vivo with MOG_37–50_ peptide were analyzed with a DELFIA® cell cytotoxicity kit (PerkinElmer), as previously described [35]. Briefly, target B lymphocytes (freshly isolated from the spleen of B6 mice) were pulsed with 10 μg/ml of MOG_37–50_ or control (gp100_25–33_) peptide for 1 h at 37 °C in culture medium. After three washes, Ag-pulsed cells were labeled (1 × 10^6^ cells/ml) with 50 μM of fluorescence-enhancing ligand bis(acetoxymethyl)2,2′:6′,2″-terpyridine-t,6″ dicarboxylate for 30 min at 37 °C. Following an additional series of washes, labeled Ag-pulsed B cells were co-cultured in 200 μl of medium at 5 × 10^3^ cell/well in 96-well flat-bottom plates with (c-Met^+^ vs. c-Met^−^) CD8^+^ T cells treated or not with HGF (30 ng/ml) at the indicated ratio. Plates were incubated for 4 h at 37 °C. After 4 h, 20 μl of culture supernatant was harvested from each well and added to separate 96-well flat-bottom plates containing 200 μl/well of 50 μM Europium solution (Aldrich Chemical) in 0.3 M acetic acid (pH 4). Plates were shaken for 15 min at room temperature, and the fluorescence of the Europium TDA (Eu) chelates formed was quantified in a time-resolved fluorometer (DELFIA 1234). All assays were performed in triplicate. Spontaneous release was determined as Eu detected in the supernatant of targets incubated in the absence of effector cells. Maximum release was determined as Eu detected in the supernatants of target cells incubated with lysis buffer instead of effectors. Percent specific lysis was calculated according to the following formula: (experimental release – spontaneous release)/(maximum release – spontaneous release) × 100

### Immunofluorescence

Cytospin spleen cells from peak disease of EAE mice (d14) were fixed with acetone. Slides were blocked for 30 min in PBS with 1% bovine serum albumin. Abs against mouse c-Met (clone; eBioclone7, eBioscience) and CD8 (clone; 53-6.7, eBioscience) were applied at 1:100. Sections were incubated overnight with both Abs, followed by several washes with PBS. Slides were mounted with ProLong Gold anti-fade reagent with DAPI (Life Technologies) and kept at 4 °C. The slides were imaged by a core facility using a Leica SP5 Axiocam system (Leica MicroImaging).

### RNA isolation and real-time quantitative PCR

RNA was prepared from MACS-sorted splenic CD8^+^ T cells at peak disease (d14) followed by separation with FACS Aria on c-Met^+^CD8^+^ or c-Met^−^CD8^+^. Total RNA extractions were performed with the Qiagen RNeasy Mini Kit and subjected to DNase I (Roche Diagnostics) digestion. Random hexamer primers (Promega, Madison, WI) and Superscript II RNase H reverse transcriptase (Invitrogen, Carlsbad, CA) were used to generate cDNA. All transcripts were quantified by real-time PCR analysis using SYBR Green as the detection agent. The PCR was performed with the 7500 Real-Time PCR System (Applied Biosystems). The following primers were used: 5 ′ to 3 ′ forward GCGCATGTTTCCTTTCTTGAG and reverse GGTCGGCCAGAACCACTTC for EOMES; forward CAGGTTCAACGACCTTCGATT and reverse GTGGTAGGTAGCCACTTGGG for RUNX; forward AGCAAGGACGGCGAATGTT and reverse GGGTGGACATATAAGCGGTTC for T-bet; forward CCCATCCCCAGGAGTCTTG and ACCATGACTAGGGGCACTGTA for GATA3; and forward CCCATCCCCAGGAGTCTTG and reverse ACCATGACTAGGGGCACTGTA for FOXP3. PCR was performed using the Taq Platinum polymerase kit (Thermo Fisher Scientific). The amplification procedure included 3 min at 94 °C followed by 35 cycles of 30 s at 94 °C for denaturation, 30 s at 55 °C for hybridization, and 1 min at 72 °C for elongation. A final 4 min at 72 °C was used for final elongation. PCR products were visualized on 1% agarose gel using Gelgreen (Ozyme) dye under ultraviolet illumination. Quantitative PCR was performed on a 7500 Real-Time PCR System with Power SYBR Green (Applied Biosystems).

### Statistical analysis

Statistical calculations were performed using the statistical analysis software GraphPad Prism, version 7.0. Data were expressed as the mean ± standard error mean (SEM), and the differences between groups were evaluated by unpaired Student’s *t* test. Intergroup comparisons were conducted by two-way analysis of variance (ANOVA) followed by Tukey’s post hoc test to determine significant differences between experimental groups. *P* values < 0.05 were considered statistically significant.

## Results

### A subpopulation of effector CD8^+^ T cells express the receptor c-Met in EAE

Recent data indicate that T cell activation in the presence of HGF induces a distinct migratory phenotype [36]. Phenotypic and functional analysis of this newly identified CTL population (c-Met^+^CTLs) showed that c-Met^+^ CTLs displayed augmented cytolytic activities compared to their c-Met^−^ CTL counterparts in vitro and in vivo [33]. We hypothesized that the c-Met signature could directly regulate CD8 effector functions in CNS demyelination.

**Fig. 1:**
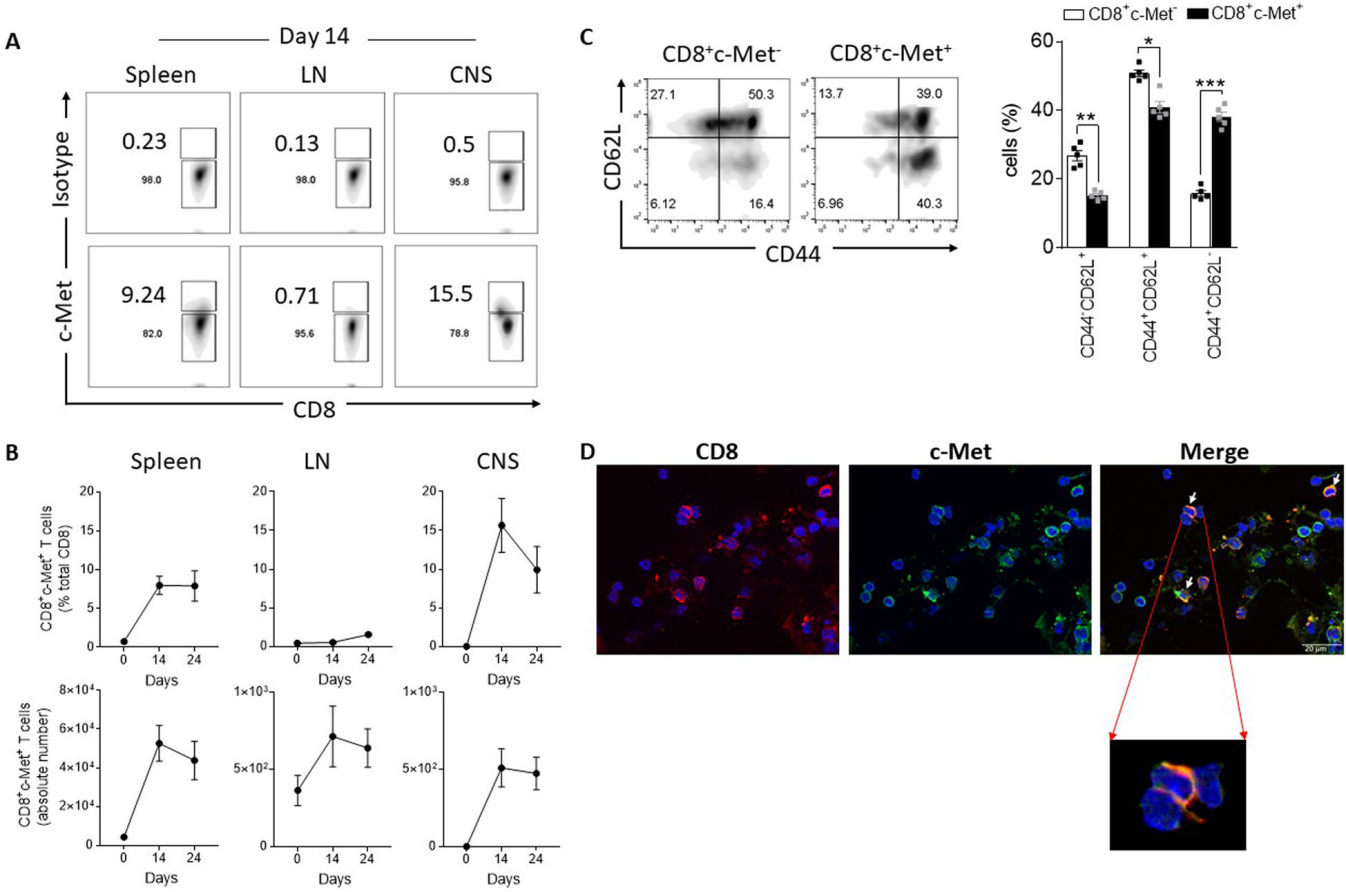
A subpopulation of effector CD8^+^ T cells expresses the c-Met receptor in EAE. MOG_35–55_ gating strategy for the identification of c-Met-expressing CD8^+^ T cells. To characterize the frequency of CD8^+^ T cells that express c-Met, live lymphocyte gated in forward (FS Lin) versus side scatter (SS Lin) dot plots were selected, followed by a subsequent gate on live (7AAD^-^) cells (not shown). CD8^+^ T lymphocytes were subsequently gated (CD45^+^CD3^+^CD8^+^ T cells). Numbers in the quadrants of the density plots (CD8 vs. c-Met) at acute phase (d14) indicate the percentage of the population CD8^+^c-Met^+^. Frequency values reflect results above the fluorescence values obtained by isotype control antibodies (**a**). Splenocytes, lymph node cells, or CNS mononuclear cells from MOG_35–55_-immunized mice were harvested and stained with CD8-APC and c-Met-FITC and analyzed by flow cytometry. Data are gated on live CD8^+^ T cells. The frequency of c-Met^+^CD8^+^ cells in each organ is depicted (**b** top panel), as is the absolute number of c-Met^+^CD8^+^ cells (**b** lower panel), based on the counts of the total number of cells obtained. Results are the averages of at least *n* = 5 mice per group per time point (representative of 3 independent experiments). **c** CD8^+^ T cells from CNS were examined by flow cytometry for expression of the activation markers CD44 and CD62L (CD44^low^CD62L^high^ naive CD8^+^ T cells; CD44^high^CD62L^low^ activated memory CD8^+^ T cells). Bars represent the mean ± standard error of values for *n* = 5 mice/group; *p ≤ 0.05, **p ≤ 0.01, and ***p ≤ 0.001 by Student’s *t* test. **d** Representative double immunofluorescent staining images for CD8 (red) and c-Met (green) of cytospin of spleen cells at peak disease. Cell nuclei were counterstained with DAPI (blue). Scale bar, 20 μm. Insets are magnified × 4.5 from the original images. The arrows indicate the area where we observed co-expression of both c-Met and CD8 markers. Comparable results were obtained with three other mice

We first assessed the expression of c-Met during the course of MOG_35–55_-induced EAE. Mice were monitored for up to 24 days (recovery phase) post-immunization (Additional file 1: Figure S1A and B). No expression of c-Met was detected in resting naive CD45^+^CD8^+^ T cells in the spleen, lymph node, or CNS (Fig. 1b). Remarkably, when compared to day 0 (pre-immunization), CD8^+^ T cells at peak disease (day 14 post-immunization) expressed significantly higher levels of c-Met receptor in the spleen and in the CNS compartment, as shown by c-Met^+^CD8^+^ frequencies and cell number graph (Fig. 1a, b). In addition, we observed an increase of c-Met^+^CD8^+^ T cell number (but not frequencies) in the LN at peak disease (versus day 0) (Fig. 1a, b). Furthermore, in examining expression of T cell activation markers at day 14 (peak disease), we found that c-Met^+^CD8^+^ T cell levels of activation were increased (CD44^high^CD62L^-^) when compared to their counterpart c-Met^−^CD8^+^ (Fig. 1c).

We next determined whether CD8^+^ T cells express c-Met receptor. As shown by immunofluorescence, c-Met expression was observed in a significant proportion of CD8^+^ cells from EAE splenocytes at peak disease (day 14) (Fig. 1d). c-Met protein level was assessed by western blotting after splenic CD8^+^ T cells sorting (d14), and the results confirm the high expression of c-Met in FACS-sorted c-Met^+^CD3^+^CD8^+^ (Additional file 1: Figure S1D). A weak c-Met expression was also observed in FACS-sorted c-Met^−^CD3^+^CD8^+^ T cells that can be explained by intracellular c-Met expression.

### Phenotypic characterization of c-Met^+^ CD8^+^ T cells ex vivo in MOG-induced EAE

**Fig. 2:**
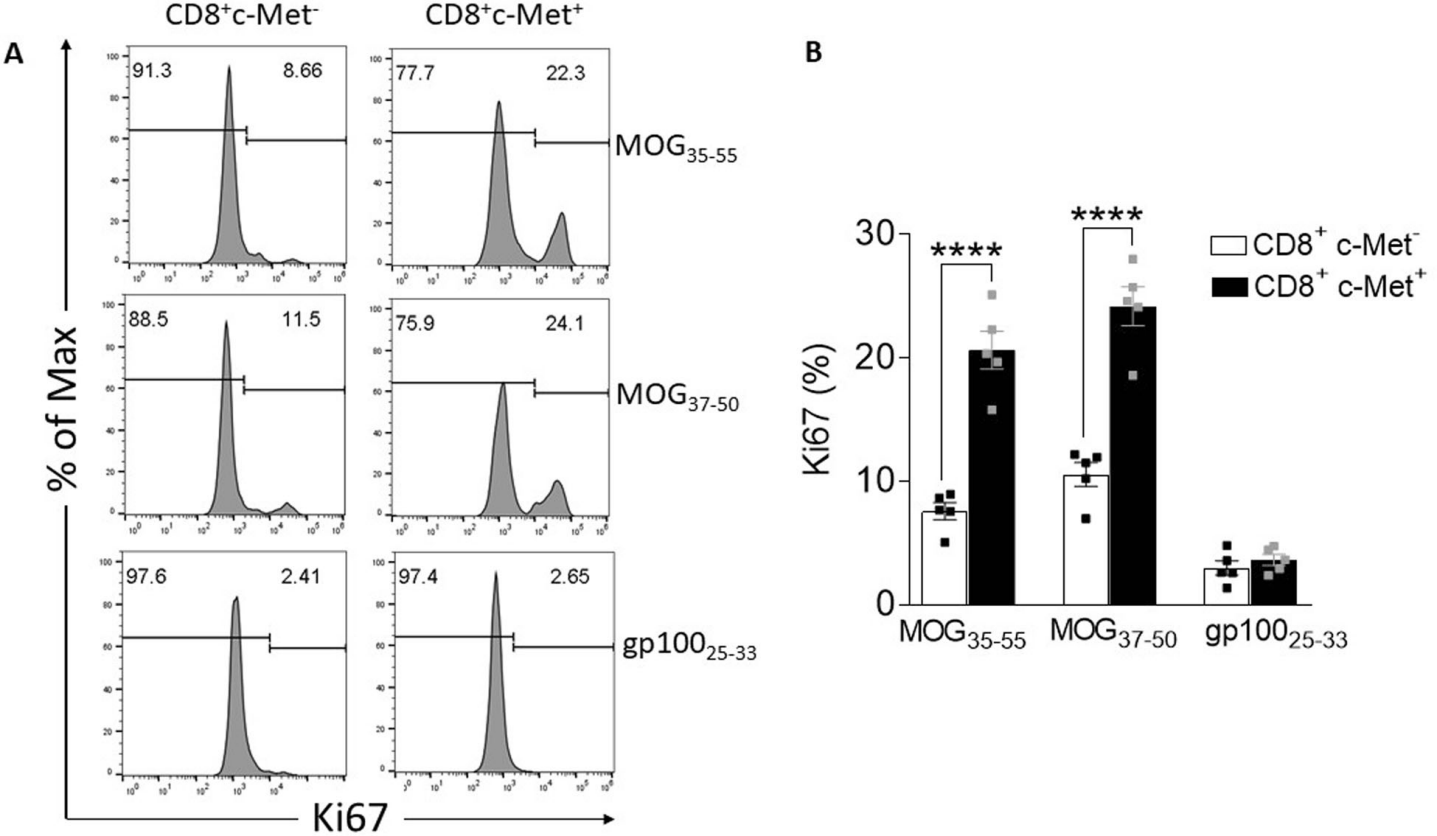
Phenotypic characterization of murine c-Met^+^ CD8^+^ T cells ex vivo in MOG-induced EAE. Splenocytes were isolated from MOG peptide-immunized mice (mean score 2.5–3.5). Cells were cultured in the presence of MOG_35–55_ or MOG_37–50_ or with gp100_25–33_ peptide control for 72 h. CD8^+^ T cells were examined by flow cytometry to quantify the frequency of Ki67-positive CD8^+^ T cells, which was determined by intracellular staining. Representative histograms are shown including quantification (**a**, **b**). Bars represent the mean ± standard error of values for *n* = 5 mice/group. ****p ≤ 0.0001 by two-way ANOVAs followed by Tukey’s post hoc test

To evaluate c-Met^+^CD8^+^ T cells in MOG-induced EAE, we analyzed recall T cell responses of CD8^+^ splenocytes from mice at peak disease (d14). Spleen cells were re-stimulated with either MOG_35–55_, MOG_37–50_, or gp100_25–33_ control peptide. We observed increased cell proliferation of c-Met^+^CD8^+^ T cells in the presence of both peptide MOG_35–55_ and MOG_37–50_ compared to their counterpart c-Met^−^CD8^+^ and the control condition (Fig. 2a, b).

### Increased granzyme B from c-Met^+^CD8^+^ T cells in EAE

**Fig. 3:**
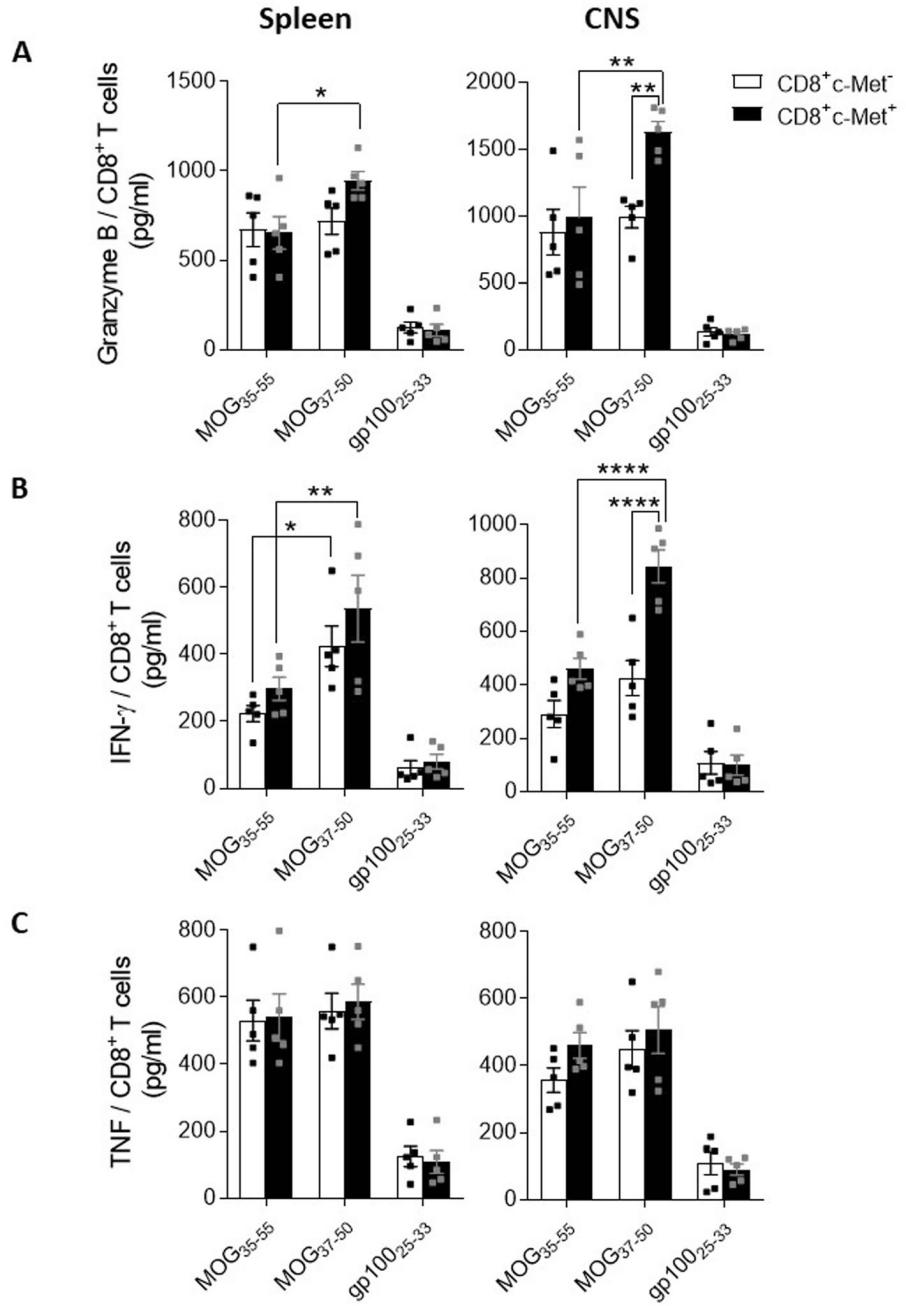
Comparison of phenotype of CTLs located in the spleen and CNS in EAE. At day 14 post-EAE induction, CD8 T cells from the spleen and from CNS-infiltrating cells were isolated and incubated with irradiated syngeneic splenocytes loaded with 1 μM of peptide (MOG_35–55_, MOG_37–50_, or gp100_25–33_ control peptide) for 48 h in the presence of 20 pg/ml IL-2. Granzyme B (**a**), IFNγ (**b**), and TNF (**c**) levels were quantified by ELISA in the cell supernatant of stimulated inflammatory T cells. *n* = 5 mice/group; data shown as mean ± standard error. *p ≤ 0.05, **p ≤ 0.01, ****p ≤ 0.0001 (two-way ANOVAs followed by Tukey’s post hoc test)

To characterize this population of CD8-expressing c-Met in MOG-induced EAE, we first isolated them from the spleen and the CNS at the peak disease (day 14). Cells were further co-cultured with irradiated syngeneic splenocytes loaded with 20 μg/ml (MOG_35–55_, MOG_37–50_, or gp100_25–33_) for 48 h in the presence of 20 ng/ml of IL-2. After re-stimulation with MOG_35–55_, we observed no difference in granzyme B and IFNγ secretion between c-Met^+^ and c-Met^−^ CD8^+^ T cells (Fig. 3a, b). Because MOG_37–50_-specific CD8^+^ T cells are generated in MOG_35–55_-induced EAE mice and MOG_37–50_ peptide retains the full ability to stimulate MOG-specific CD8^+^ T cells ex vivo [14], we further examined the stimulation of c-Met^+^CD8^+^ T cells from both the spleen and CNS with MOG_37–50_ peptide. c-Met^+^CD8^+^ T cells re-stimulated ex vivo with MOG_37–50_ peptide released significantly higher levels of granzyme in both the spleen and CNS, and c-Met^+^CD8^+^ T cells from the CNS but not from the spleen released increased levels of IFNγ (Fig. 3b). Finally, we found no difference in TNF secretion by either CD8^+^ T cell population (Met^+^ vs Met-) in response to encephalitogenic peptides (MOG_35–55_ or MOG_37–50_) (Fig. 3c). These data suggest that the MOG_37–50_ peptide generates c-Met^+^CD8^+^ T cell-specific responses and increased granzyme B and IFNγ release ex vivo in MOG-induced EAE.

### Expression modulation of CD8^+^-specific lineage markers by HGF

**Fig. 4:**
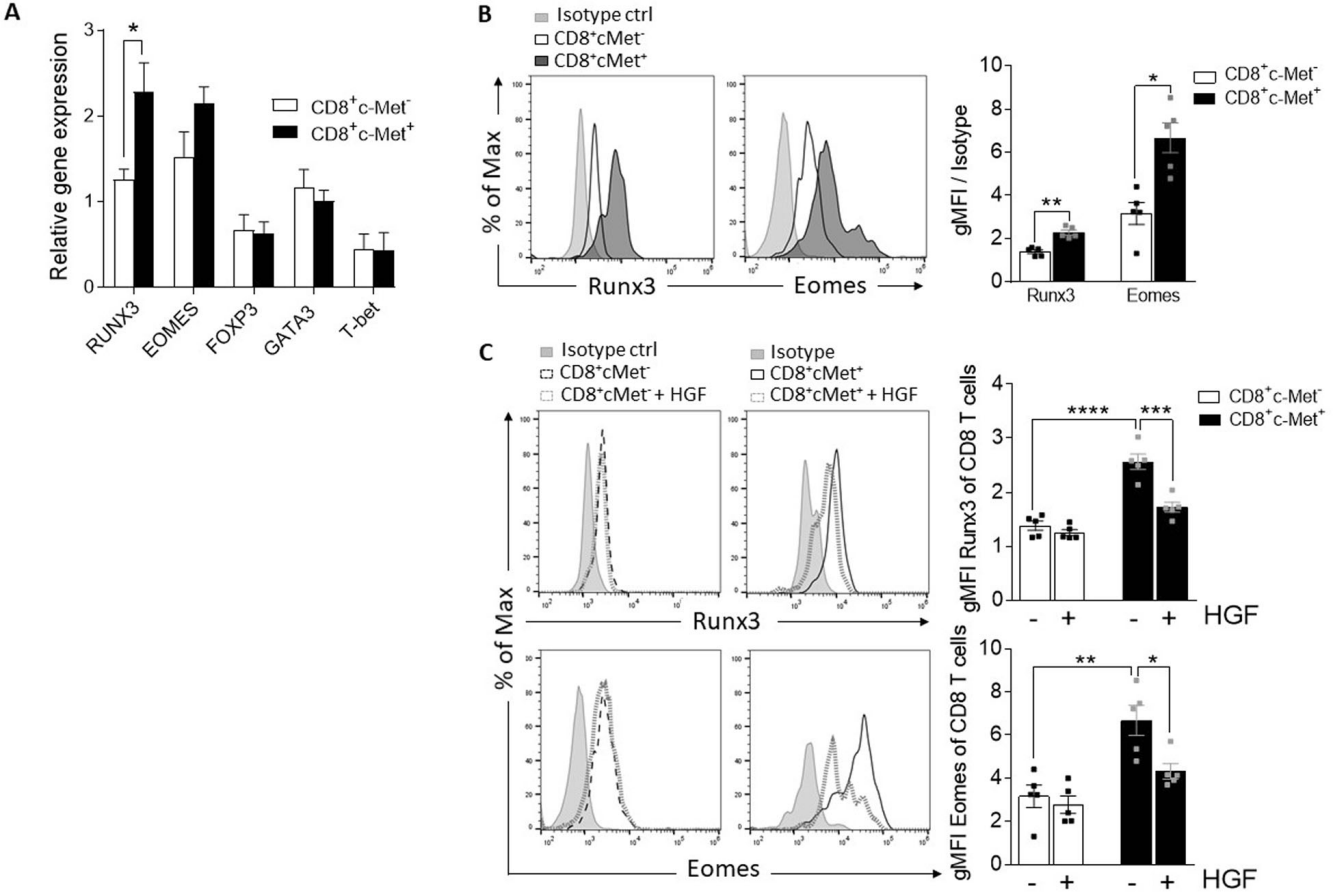
Ex vivo gene and protein characterization of c-Met^+^ versus c-Met^-^CD8^+^ T cells. **a** Relative expression of EOMES, RUNX3, FOXP3, GATA3, and T-bet mRNA in FACS-sorted spleen cells of peak disease c-Met^+^ CD3^+^ CD8^+^ vs. c-Met^−^ CD3^+^ CD8^+^ T from MOG_35–55_-induced EAE mice, as measured by quantitative RT-PCR, representative of *n* = 3 mice; ∗*p* ≤ 0.05 (unpaired, 2-tailed Student’s *t* test). **b** Representative histogram and average MFI (bar graph) of Runx3 and Eomes in EAE c-Met^+^ vs. c-Met^−^ CD3^+^CD8^+^ T cells, representative of *n* = 5 mice. **c** Eomes and Runx3 protein levels in spleen cells at peak disease (d14) c-Met^+^CD8^+^ vs. c-Met^−^ CD8^+^ T cells from MOG-induced EAE mice treated with either vehicle or HGF (30 ng/ml for 48 h) in the presence of IL-2; representative histograms are shown. Measurement of Eomes and Runx3 gMFI compared to isotype control of each condition (**b**, **c**). Data are representative of *n* = 5 mice. *p ≤ 0.05, **p ≤ 0.01 (unpaired, 2-tailed Student’s *t* test for **b**). Data from **c** are presented as mean ± SEM and were analyzed using the two-way ANOVA followed by Tukey post hoc comparisons. *p ≤ 0.05, **p ≤ 0.01, ***p ≤ 0.001, ****p ≤ 0.0001

Our previous data indicated that cytotoxic T cells expressing c-Met (c-Met^+^CTLs) are highly cytotoxic compared to their counterpart c-Met^−^CTLs [33], and another group showed that T cell activation in the presence of HGF induces a distinct migratory phenotype [36]. We hypothesized that c-Met protein expression in CD8^+^ T cells could directly regulate their gene and protein expression. To address this hypothesis, we first assessed the master regulator genes (RUNX3, EOMES, FOXP3, GATA3, and T-bet). mRNAs were extracted from sorted spleen c-Met^+^ vs. c-Met^−^ CD8^+^ T cells at peak disease (d14) in MOG-induced EAE. RT-PCR data showed that c-Met^+^CD8^+^ T cells expressed higher levels of RUNX3 and EOMES compared to c-Met^−^CD8^+^ T cells; however, we observed no difference in the expression of Th1, Th2, or Treg-specific regulator genes such as T-bet, GATA3, and FOXP3, respectively (Fig. 4a). In flow cytometry assessment of Runx3 and Eomes protein expression, splenic c-Met^+^CD8^+^ T cells expressed higher levels of Runx3 and Eomes protein compared to c-Met^−^CD8^+^ T cells (Fig. 4b).

We next assessed the effects of HGF treatment on CD8^+^ T cells expressing c-Met receptor. Spleen cells from peak disease (d14) were treated with HGF (30 ng/ml) ex vivo for 48 h in the presence of IL-2. We found that HGF-treated c-Met^+^CD8^+^ T cells expressed significantly lower intracellular levels of Eomes and Runx3 protein compared with the untreated condition (Fig. 4c). As expected, HGF did not change the expression of Eomes and Runx3 in c-Met^−^CD8^+^ T cells (Fig. 4c).

### Modulation of c-Met^+^CD8^+^ T cell phenotype and function by HGF

**Fig. 5:**
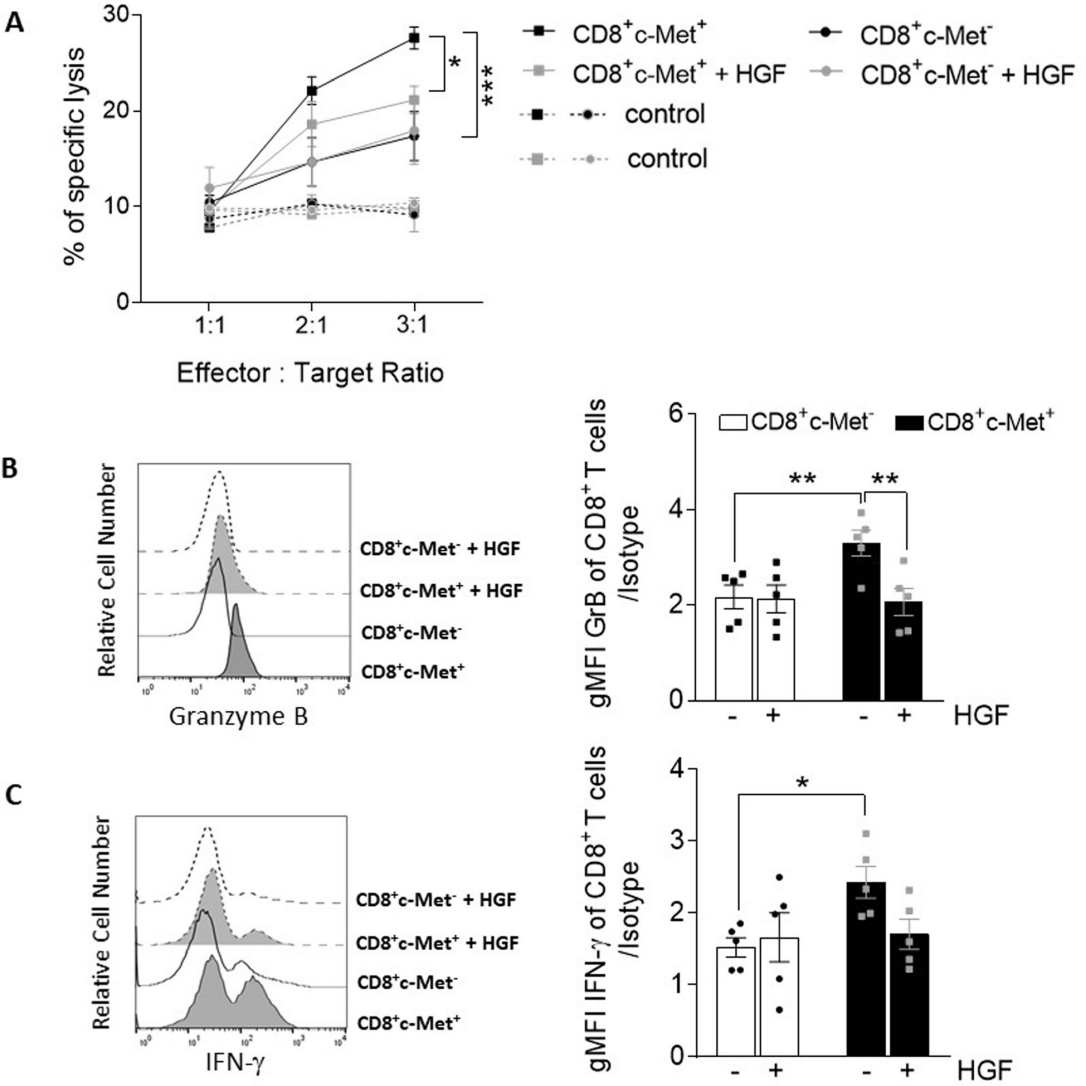
HGF modulates the phenotype and function of c-Met^+^CD8^+^ T cells. **a** Cytolytic activity of CD8^+^ obtained from MOG_35–55_-induced EAE (score 2.5–3.5) stimulated ex vivo with MOG_37–50_ peptide for 48 h. Various effector/target (E/T) ratios were tested for killing of syngeneic B cells pulsed with MOG_37–50_ peptide (squares) or control gp100_25–35_ peptide (circles). Values represent the mean ± standard error of *n* = 3 mice per group, carried out in triplicate. Representative histogram and average MFI (bar graph) of granzyme B (**b**) and IFNγ (**c**) of c-Met^+^ vs. c-Met^−^ CD3^+^CD8^+^ T cells from EAE mice (d14) treated ex vivo with HGF (30 ng/ml for 48 h) or untreated as vehicle; *n* = 5 mice/group. Data are presented as mean ± SEM and were analyzed using the two-way ANOVA followed by Tukey's post hoc comparisons. *p ≤ 0.05, **p ≤ 0.01

We next assessed the effect of HGF treatment on ex vivo c-Met^+^CD8^+^ T cells isolated at d14 from the spleen. T cells were stimulated with MOG_37–50_ peptide for 48 h before co-culture with the target cells loaded with the same peptide. HGF treatment significantly blunted the ex vivo cytotoxic function of c-Met^+^CD8^+^ T cells but had no effect on the killing ability of c-Met^−^CD8^+^ (Fig. 5a). In addition, HGF treatment decreased the capacity of bulk (i.e., c-Met^+^ and c-Met^−^) CD8^+^ T cells isolated from the spleen of EAE mice to produce granzyme B (Fig. 5b) and weak decrease of IFNγ, though not significant (Fig. 5c). As expected, HGF treatment did not change the expression profile of cytotoxic effector molecules in c-Met^−^CD8^+^ T cells (Fig. 5b, c). Taken together, these results show that HGF down-modulates the cytolytic function of c-Met^+^ CTLs through regulation of cytotoxic effector molecule expression such as granzyme B. These findings are in agreement with our previous data [33].

## Discussion

HGF acts by binding to the HGF tyrosine kinase receptor, c-Met [37]. Both are expressed in brain-resident cells including neurons [38], mature oligodendrocytes [39, 40], oligodendrocyte progenitor cells [41, 42], and microglia [43]. In addition to its neuroprotective effect, HGF blunts inflammation in a variety of inflammatory T cell-mediated disease models, suggesting that it suppresses a common inflammatory process. MOG-induced EAE is a model of MS mediated by encephalitogenic CD4^+^ T cell responses and characterized by demyelination and axonal loss [44]. We have previously demonstrated that overexpression of neuronal HGF attenuates disease progression in this model, partly via anti-inflammatory signals [26]. Using this MS model, we established that HGF exerts an anti-inflammatory effect through the generation of tolerogenic dendritic cells and the subsequent suppression of autoreactive peripheral Th1 and Th17 cells, leading to reduced CD4^+^ T cell-mediated CNS injury. Whether HGF modulates cell-mediated immunity driven by MHC class I-restricted CD8^+^ T cells remained however unclear.

In addition, we recently demonstrated in an established model of murine CTL-mediated killing that HGF treatment decreases levels of effector CTL molecules (IFNγ, TNF, perforin, granzyme B), as well as expression of CD107a, a marker of CD8^+^ T cell degranulation [35]. Based on these findings, we suggested that expression of c-Met on CD8^+^ T infiltrating cells may represent an additional pathway that limits the function of these T cells in the tumor microenvironment and ultimately favors tumor outgrowth [33].

Multiple observations support the idea that CD8^+^ T cells, in addition to pathogenic CD4^+^ T cells, are involved in the pathogenesis of CNS autoimmunity as active contributors to the development of neuroinflammation. In MS, CD8^+^ T cells outnumber by far CD4^+^ T cells in both acute and chronic inflammatory lesions. In CNS, although CD4^+^ T cells show a primarily perivascular distribution, CD8^+^ T cells can be detected in the parenchyma [1, 2]. Although normally poorly expressed, MHC class I molecules are highly expressed within the MS lesion on astrocytes, oligodendrocytes, and neurons, suggesting that CD8^+^ T cells could be directly engaging these cell types [45, 46].

In this report, with the widely used MOG_35–55_ EAE mouse model of MS, we demonstrate that a subgroup of the encephalitogenic CD8^+^ T cell population expresses c-Met receptor. This c-Met^+^CD8^+^ T cell population significantly increased at the time of peak clinical disease and decreased in the recovery phase, similarly to the encephalitogenic MOG_37–50_-specific CD8^+^ T cell response during EAE [14]. These c-Met^+^CD8^+^ T cells have a higher level of activation compared to c-Met^−^CD8^+^ T cells, which agrees with our previous observations related to murine c-Met^+^CTLs [33, 35]. Using ex vivo and in vitro strategies, we report here that c-Met^+^ and c-Met-CD8^+^ T cells in MOG-induced EAE exhibit a different functional phenotype and that HGF treatment downregulates the effector functions of c-Met^+^CD8^+^ T cells, as described in our previous studies [33, 35]. We have shown the capacity of HGF to reduce the encephalitogenic property of this subset of CTLs and suggest that expression of c-Met in CD8^+^ T cells may have a role in the causality of CNS pathology.

Our results are consistent with those of others demonstrating pathogenic potential of effector CD8^+^ T cells in a myelin basic protein model of EAE in C3HeB mice [9]. The in vitro killing assay revealed a higher capacity of EAE-isolated c-Met^+^CD8^+^ T cells to lyse their specific target cells compared to c-Met^−^CD8^+^ T cells. These data are in line with findings on oligodendrocyte-specific autoreactive CD8^+^ T cells that can lyse oligodendrocytes in vitro [17]. CD8^+^ T cells induce demyelination through secretion of cytolytic factors, such as granzyme B, IFNγ, and lymphotoxin [16–18]. These data support our current results showing that c-Met^+^CD8^+^ T cells produce a relatively higher level of granzyme B and IFNγ. The implication of these observations is that expression of c-Met on CD8^+^ T cells in EAE may represent an additional pathway that explains the function of these T cells in the neuroinflammation microenvironment, ultimately favoring disease aggravation.

We found that HGF potently inhibits c-Met^+^CD8^+^ T cell-mediated killing through interference with decreases in granzyme B and IFNγ, suggestive of a direct effect of HGF on dual granzyme B/IFNγ-based CTL-mediated cytotoxicity. Because the perforin/granzyme B pathway has been implicated in potential mechanisms of oligodendrocyte and/or axonal injury and demyelination in MS [7], our findings together suggest that increased HGF may reduce CTL effector function in CTL-mediated human autoimmune disorders of the CNS.

Important unanswered questions now arise from the current findings including which mechanisms are involved in the generation of c-Met^+^CD8^+^ T cells and how does HGF regulate this new population? The demonstration of the inhibitory effect of HGF should be further confirmed with c-Met blocker experiments. In addition, our data indicate that although c-Met expression is undetectable in naive CD8^+^ T cells in non-immunized mice, it characterizes a significant proportion of CD8^+^ T cells at peak disease and remains during the recovery phase. This pattern suggests that the population of c-Met^+^CD8^+^ T cells arise from pathologic changes associated with EAE development, as shown in tumor studies [33].

## Conclusions

Our findings highlight the role of CD8^+^ T cell subpopulation expressing the c-Met receptor in a model of CNS autoimmunity. HGF may thus be effective in preventing inflammatory neurodegenerative disease via its immunosuppressive role on CD8^+^ T cells. Further studies in both EAE animal models and in MS are needed to provide a better understanding of the role of the HGF/c-Met axis in modulation of neuroinflammation.

## Supplementary information

**Additional file 1: Figure S1.** MOG-induced EAE clinical score. (A) MOG_35–55_ induces EAE in B6 mice. EAE was induced by immunization with 200 μg of MOG_35–55_, emulsified in CFA on days 0. Mice also received 300 ng of pertussis toxin intravenously on days 0 and 2. EAE disease severity was followed, and incidence is shown in (B). (C) The forward scatter of both populations’ c-Met^+^ vs c-Met^-^ CD8^+^ cells are represented. (D) Protein expression levels of c-Met by CD8^+^ T cells in EAE at day 14. Lines 1, 2, 3 correspond respectively to: 1) bulk splenic CD8^+^ T cells enriched by negative selection using μbeads, 2) FACS-sorted (EAE d14) c-Met^+^CD3^+^ CD8^+^ T cells and 3) FACS-sorted (EAE d14) c-Met^-^CD3^+^ CD8^+^ T cells, as measured by Western blot (n=3 mice). Purified CD8+ T cells were homogenized using a polytron in lysis buffer (50 mM Tris–HCl [pH 7.5], 250 mM NaCl, 1% Triton X‐100, 1 mM EDTA, and 1 mM DTT) containing complete protease inhibitors (Roche). Equal amounts (20 μg) of total protein from each sample were transferred to a 15% sodium dodecyl sulfate (SDS)–polyacrylamide gel and blotted onto anImmobilon‐P polyvinylidene difluoride (PVDF) membrane (Millipore). Expression levels of c‐Met were detected using properly diluted (1:100) mouse monoclonal anti‐c‐Met Ab (clone 3i20,Abcam), followed by a peroxidase‐conjugated secondary Ab to mouse IgG1 (eBioscience), and then visualized using chemiluminescence (Supersignal; Pierce). The blot was also probed with mouse monoclonal anti‐β‐actine (clone 15G5A11/E2) as a loading control (Sigma‐Aldrich).

## Data Availability

The datasets used and/or analyzed during the current study are available from the corresponding author on reasonable request.

## Abbreviations

CFA: Complete Freund’s adjuvant
CNS: Central nervous system
CTL: Cytotoxic T lymphocytes
EAE: Experimental autoimmune encephalomyelitis
EOMES: Eomesodermin
FOXP3: Forkhead box P3
GATA3: Trans-acting T cell-specific transcription factor
HGF: Hepatocyte growth factor
i.p: Intraperitoneal
i.v: Intravenous
IFN: Interferon
LN: Lymph node
MOG: Myelin oligodendrocyte glycoprotein
MS: Multiple sclerosis
PMA: Phorbol myristate acetate
RUNX3: Runt-related transcription factor 3
T-bet: T-box transcription factor
Th: T helper
TNF: Tumor necrosis factor
Treg: T regulatory
