# Supplementary figures and images for: c-Met is expressed by highly autoreactive encephalitogenic CD8+ cells
Source: J Neuroinflammation. 2020 Feb 19;17:68. doi: 10.1186/s12974-019-1676-0 (PMC7031922; doi:10.1186/s12974-019-1676-0)

Supplementary figure 1

**A**

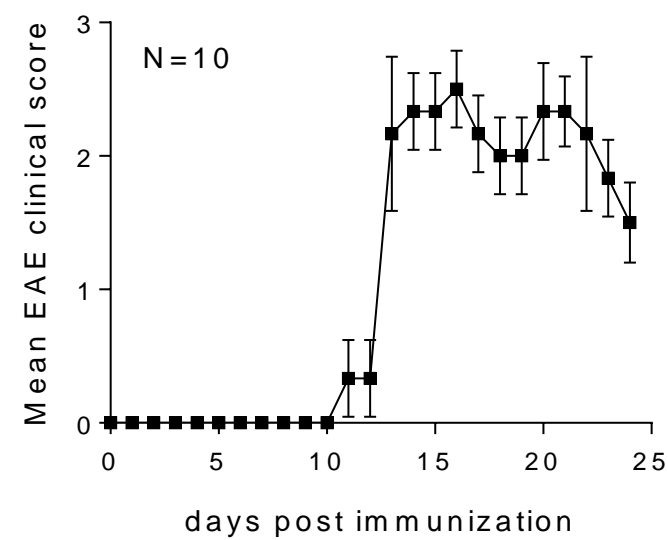

**B**

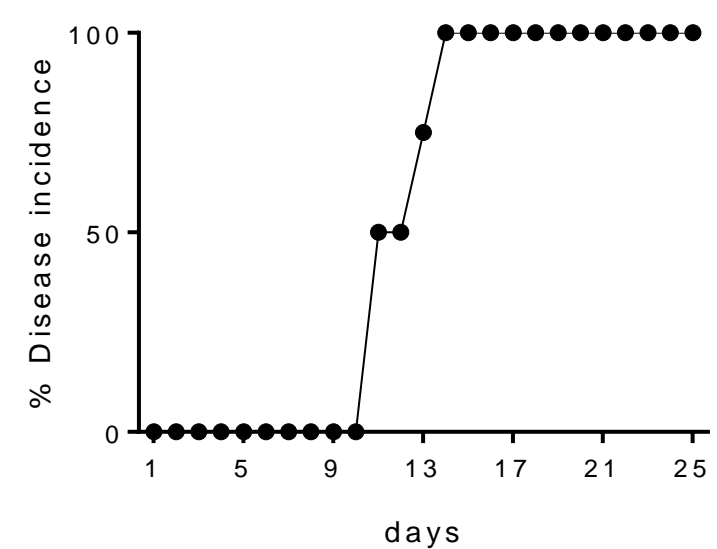

**C**

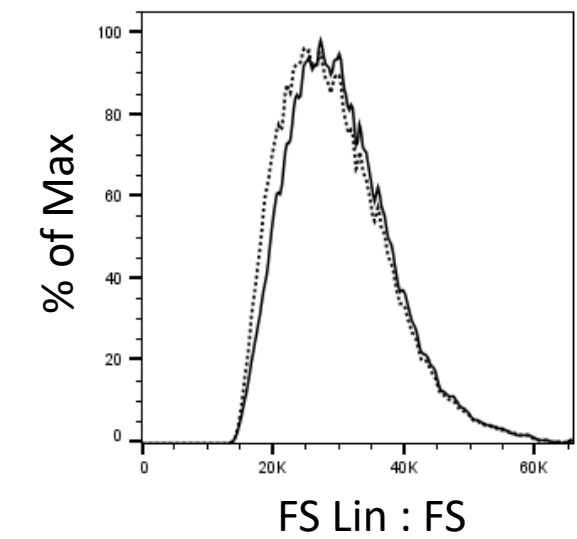

**D**

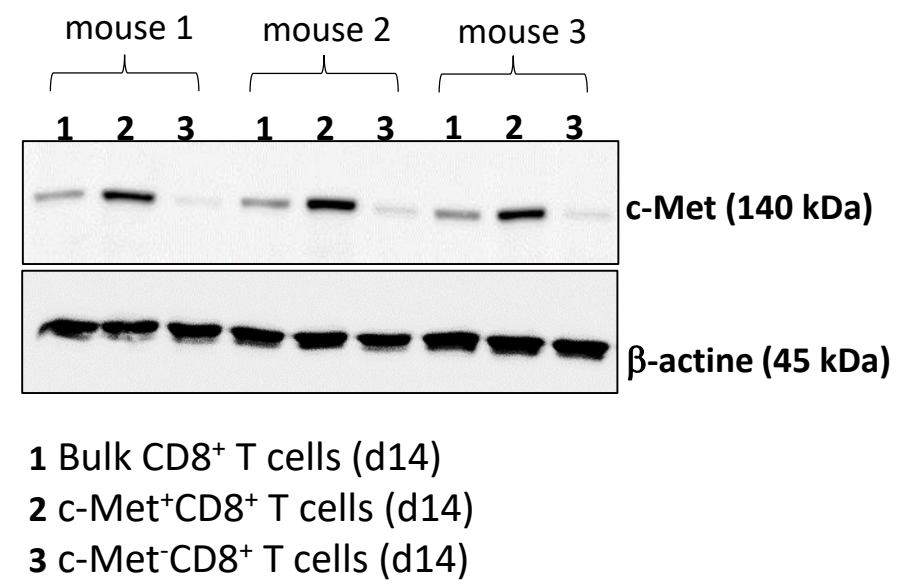

Supplement: Supplementary file 1 — Additional file 1: Figure S1. MOG-induced EAE clinical score. (A) MOG35–55 induces EAE in B6 mice. EAE was induced by immunization with 200 μg of MOG35–55, emulsified in CFA on days 0. Mice also received 300 ng of pertussis toxin intravenously on days 0 and 2. EAE disease severity was followed, and incidence is shown in (B). (C) The forward scatter of both populations’ c-Met+ vs c-Met- CD8+ cells are represented. (D) Protein expression levels of c-Met by CD8+ T cells in EAE at day 14. Lines 1, 2, 3 correspond respectively to: 1) bulk splenic CD8+ T cells enriched by negative selection using μbeads, 2) FACS-sorted (EAE d14) c-Met+CD3+ CD8+ T cells and 3) FACS-sorted (EAE d14) c-Met-CD3+ CD8+ T cells, as measured by Western blot (n=3 mice). Purified CD8+ T cells were homogenized using a polytron in lysis buffer (50 mM Tris–HCl [pH 7.5], 250 mM NaCl, 1% Triton X‐100, 1 mM EDTA, and 1 mM DTT) containing complete protease inhibitors (Roche). Equal amounts (20 μg) of total protein from each sample were transferred to a 15% sodium dodecyl sulfate (SDS)–polyacrylamide gel and blotted onto anImmobilon‐P polyvinylidene difluoride (PVDF) membrane (Millipore). Expression levels of c‐Met were detected using properly diluted (1:100) mouse monoclonal anti‐c‐Met Ab (clone 3i20,Abcam), followed by a peroxidase‐conjugated secondary Ab to mouse IgG1 (eBioscience), and then visualized using chemiluminescence (Supersignal; Pierce). The blot was also probed with mouse monoclonal anti‐β‐actine (clone 15G5A11/E2) as a loading control (Sigma‐Aldrich). [file 12974_2019_1676_MOESM1_ESM.pdf]
